# Impact of preoperative antithrombotic therapy on blood management after implantation of primary total knee arthroplasty

**DOI:** 10.1038/srep30924

**Published:** 2016-08-04

**Authors:** Lukas Leitner, Ewald Musser, Norbert Kastner, Jörg Friesenbichler, Daniela Hirzberger, Roman Radl, Andreas Leithner, Patrick Sadoghi

**Affiliations:** 1 Department of Orthopaedic Surgery, Medical University of Graz, Austria

## Abstract

Red blood cell concentrates (RCC) substitution after total knee arthroplasty (TKA) is correlated with multifold of complications and an independent predictor for higher postoperative mortality. TKA is mainly performed in elderly patients with pre-existing polymorbidity, often requiring permanent preoperative antithrombotic therapy (PAT). The aim of this retrospective analysis was to investigate the impact of demand for PAT on inpatient blood management in patients undergoing TKA. In this study 200 patients were retrospectively evaluated after TKA for differences between PAT and non-PAT regarding demographic parameters, preoperative ASA score > 2, duration of operation, pre-, and intraoperative hemoglobin level, and postoperative parameters including amount of wound drainage, RCC requirement, and inpatient time. In a multivariate logistic regression analysis the independent influences of PAT, demographic parameters, ASA score > 2, and duration of the operation on RCC demand following TKA were analyzed. Patients with PAT were significantly older, more often had an ASA > 2 at surgery, needed a higher number of RCCs units and more frequently and had lower perioperative hemoglobin levels. Multivariate logistic regression revealed PAT was an independent predictor for RCC requirement. PAT patients are more likely to require RCC following TKA and should be accurately monitored with respect to postoperative blood loss.

Primary total knee arthroplasty (TKA) belongs to the large standard procedures in orthopaedic surgery and is therefore correlated with severe complications^1^. Substantial blood loss is amongst the most common complications following TKA, often requiring transfusion of red blood cell concentrates (RCC) in the post-operative period^2^. Earlier determined risk factors for RCC substitution after TKA are age, preoperative anemia, female sex, body mass index (BMI) <30, and ASA classification >2^3^,^4^. After TKA a transfusion rate between 18.3% and 67% has been described^3^,^5^,^6^. Transfusion strategy following large orthopaedic procedures is discussed controversially, since in a large multi-center study (n = 2016) a liberal transfusion strategy did neither reduce mortality nor improve recovery after hip-fracture surgery^7^. Furthermore, although blood transfusion is considered a safe treatment today, some authors found a correlation between RCC substitution and severe complications like infection, pneumonia, thromboembolism, prolonged hospital stay, and death in patients after knee or hip arthroplasty^8^,^9^,^10^.

TKA is mainly performed in elderly patients leading to an increasing number of primary TKAs in western countries^11^. The higher rate of co-morbidity in elderly patients leads to a correlation between age and the rate of requirement for permanent or long-time antithrombotic therapy^12^. The rate of patients, requiring antithrombotic therapy is steadily increasing^13^. It remains unclear if such long-term preoperative antithrombotic therapy (PAT) could be an additional independent risk factor for RCC transfusion after TKA and lead to subsequent complications.

Therefore, the aim of this retrospective analysis was to investigate the impact of PAT on inpatient blood management in patients undergoing primary total knee arthroplasty (TKA) for osteoarthritis of the knee joint. Our hypothesis was that patients with PAT would need red cell concentrate substitution more frequently and to a higher extent.

## Methods

### Study population and data

After Institutional Review Board approval was obtained, 200 patients who underwent TKA in regional and/or general anesthesia between 2002 and 2011 (female: 63%, age: 71.4 ± 7.9 years), were selected via our hospital database system for this retrospective data analysis. Main inclusion criteria were that data on RCC demand, number and wound drainage had been recorded. In detail we retrieved data including demographic characteristics (age, sex, BMI), preoperative and perioperative antithrombotic therapy (substance, duration of intake before surgery, last intake before surgery), data affecting the operation (duration, pre- and intraoperative hemoglobin (Hb)-level) and postoperative management (wound drainage on the first post-OP day, RCC demand and number of units on surgery day to 6^th^ post-OP day, inpatient time). Exclusion criteria were revision surgery or syndromic inherited coagulation disorders. Anesthesiology classified the patients into ASA-groups prior to surgery. Moreover, they were divided in PAT and non-PAT patients depending on whether a permanent demand for oral or subcutaneous antithrombotic therapy had existed before operation or not.

### Institutional perioperative anticoagulation and transfusion policy

For PAT patients, switch of antithrombotic therapy to Enoxaparin, which is used as antithrombotic perioperative prophylaxis, is recommended 7–10d prior surgery and kept during inpatient stay. The transfusion practices of our institution are in accordance with the guidelines of the Austrian society of transfusion medicine (ÖGBT), in short recommending a transfusion ‘nearly always’ at a Hb < 6 g/l, ‘contemplate’ at a Hb 6–8 g/l and ‘usually not’ at a Hb 8–10 g/l. Additionally, until 2010, patients older than 80 years were prevented from having a hemoglobin level <10 g/dl by liberal RCC transfusion.

### Statistical methods

We compared demographic characteristics, comorbidities, and intra and post-operative characteristics of PAT and non-PAT patients, and patients with or without RCC demand after arthroplasty. Statistical analysis was performed using chi-squared test for comparison of categorical parameters, t-test for comparison of continuous normally distributed parameters and Spearman’s correlation coefficient for calculation of correlations. Multivariate logistic regression analysis was performed to identify independent factors, predicting RCC demand after TKA as earlier described by Hart *et al*.^3^. A p-value < 0.05 was considered to be statistically significant.

## Results

Out of our total patient population, 41% required PAT due to concomitant non-surgical diseases before TKA was planned. Most patients received PAT due to cardiovascular disease (CVD, 35.7%) followed by cardiac arrhythmia (22.0%) and history of thrombosis or pulmonic embolism (17.4%), the most common antithrombotic agent in PAT patients was aspirin (53.7%) followed by phenprocoumon (37.8%), average length of use before operation was 51.9 ± 34.1 months and the time since last dose was 9.5 ± 5.1 days (Table 1). 20.5% of the patients included in this study were administered at least one RCC unit after TKA. Age was significantly correlated with the number of RCC units needed (r = 0.254; p < 0.001). PAT and non-PAT patients are compared in detail in (Table 2). Out of total 84 RCC transfusions indicated within this study, the majority was given on the first day after operation (27.4%), followed by surgery day (17.9%) and a decreasing tendency in the following days.

Patients with CVD, cardiac arrhythmia, history of myocardial infarction (MI) or stroke/transient ischemic attack (TIA), which all might lead to more liberal RCC transfusion, had no significant increased transfusion rate (Table 1).

Patients with preoperative antithrombotic therapy were significantly older at the time of surgery (p = 0.002) and were classified ASA > 2 more often (p < 0.001). PAT patients did not significantly differ in preoperative Hb-levels but had a significantly lower intraoperative Hb-level (p = 0.002) and needed RCC after surgery more frequently (p < 0.001), resulting in a higher number of transfused RCC units per patient on average (p = 0.001). We found no significant correlation between lowest intraoperative Hb-level and duration of operation in all patients and PAT and non- PAT patients subgroups. PAT patients were put on significant higher Enoxaparin dose before operation (p < 0.001) but had no significant increase in amount of wound drainage on first postoperative day. Duration of hospital stay did not differ significantly between the two groups (Table 2).

We further found that patients, treated with RCC (RCC-patients) also differed significantly from the rest of our patient population in other respects (Table 3): RCC-patients were older at the time of surgery (p = 0.002), their ratio of female patients (p = 0.025) and ASA > 2 patients (p = 0.05) was higher in RCC-patients and a significantly higher number of them received PAT (p < 0.001). Furthermore RCC-patients had a significantly lower preoperative (p < 0.001) and intraoperative (p < 0.001) Hb-level and longer inpatient time (p = 0.042).

We performed a subsequent multivariate logistic regression analysis, in order to identify independent risk factors for demand of RCC transfusion, where PAT was identified as a significant independent risk factor for postoperative RCC transfusion (OR 1.17, p = 0.005) (Table 4). Other significant independent risk predicting factors for postoperative RCC transfusion were age at surgery (OR 1.01 per year, p = 0.014) and preoperative Hb (OR 0.9 per mg/ml, p < 0.001). BMI, sex, duration of operation, and a preoperative ASA > 2 were no significant independent risk factors in our study population (Table 4). In our study population, CVD, cardiac arrhythmia, history of MI, history of stroke/TIA, which might lead to more liberal RCC transfusion, or Enoxaparin dose before operation were no significant independent risk factors for RCC demand as against PAT when added to the multivariate logistic regression model (data not shown).

## Discussion

The aim of this retrospective analysis was to investigate the impact of PAT on inpatient blood management in patients undergoing TKA for osteoarthritis of the knee joint.

According to our hypothesis patients with PAT would need red cell concentrate substitution more frequently and to a higher extent. The most important finding of this study was that in our study population PAT was a new, significant risk factor for RCC demand after TKA, independent from earlier published risk factors^3^,^4^.

Nowadays PAT is an increasing condition in the overall population^13^ and also in patients undergoing TKA. 41% of our randomly selected study population required antithrombotic therapy prior to surgery. This high rate could be a consequence of the relatively high age of our study population (71.4 ± 8 years) and also due to the fact that this single-center study was conducted in a maximum care hospital with an increased high-risk patient rate. The interpretation, that high age of our study population is causal for the high rate of PAT patients, seems to be underlined by our finding, that PAT patients were significantly older even within our study population (Table 2).

In our study population 20.5% of patients required RCC after TKA, which is similar to transfusion rates after TKA in earlier published data^3^. In our patient population PAT was a significant independent risk factor (adjusted for sex, age, BMI, pre and intraoperative Hb, duration of the operation and ASA > 2) resulting in an 17% increase in demand for postoperative RCC substitution (Table 4). Addressing the high variety of health conditions leading to indication for permanent antithrombotic therapy^14^, we presume that this is because PAT-patients are a risk group for hereditary or acquired disorders of the coagulation system. This interpretation is underlined by our calculation that PAT, but not a specific condition leading to indication for PAT (CVD, MI, cardiac arrhythmia, history of stroke/TIA and others) as such, presented statistically significant risk factors for RCC substitution (Table 1).

Starting from a similar preoperative Hb level, PAT patients had a significantly decreased value of lowest perioperative Hb but no significantly increased amount of wound secretion on first postoperative day (Table 2). We hypothesize that the increased demand for RCC in PAT patients results from increased intraoperative blood loss, which was also assumed by other authors^2^, but not recorded within this study. In this context, the impact of increasingly used tranexamic acid and autologous transfusion and their potency in reducing blood loss in PAT patients would be interesting but was not evaluated in this study^15^.

In addition, a significantly longer hospital stay (p < 0.001) was explored in our RCC patients. This is in line with our results and previous investigators who found longer hospital stay for patients who received more blood transfusions in total knee arthroplasty^16^,^17^. Of note, PAT patients in our study population had a higher rate of preoperative ASA > 2 (‘severe systemic disease’ or worse) but preoperative ASA > 2 revealed no significant independent risk factor for postoperative RCC demand in our multivariate regression model (Table 4). This also could, as mentioned above, indicate that PAT patients may have needed RCCs more often due to their coagulopathy and not due to their higher ASA and more severe systemic disease status.

Lower preoperative Hb was another independent risk factor for RCC demand after TKA (Table 4), as preoperative anaemia was earlier reported as significant risk factor for receiving a transfusion after total knee and hip arthroplasty^3^ and also in other interventions as aortic valve implantation^18^. In this context, although female study participants needed more RCC units (p = 0.049) and were substituted RCC more often (p = 0.025) as reported in earlier studies^3^, female sex itself was no significant independent risk factor for RCC demand (Table 4). This might be conditioned by significantly lower pre-operative baseline Hb values we explored in women (15.1 v.s. 13.4 mg/ml; p < 0.001), which was also reported in earlier studies^19^, as we presume.

We hereby want to underline the following limitations of our work:

One aspect of the transfusion strategy in our hospital was changed in the period our study was set: Until 2010, patients older than 80 years were prevented from having a hemoglobin level <10 g/dl by liberal RCC transfusion, a strategy proven ineffective in later investigations^7^,^20^. Twenty-two of the patients included in this study were older than 80 years, the transfusion rate in this group was 50%, 9 of those were PAT patients, which could have presented a meaningful bias in our analysis. However, we found that the exclusion of these 22 patients from our calculations sustained a significant higher RCC transfusion rate in PAT patients (p < 0.001) and due to the small amount of patients, this does not present a meaningful bias of our analysis.

Although the findings from our study population are widely in line with earlier published results, the assertiveness of this study is limited, caused by relatively small study population and analyzing together very different regimes.

## Conclusion

Findings from our study population indicate that PAT is a significant independent risk factor for RCC demand after TKA. This is relevant, because other authors could show that the number of PAT patients is increasing and RCC demand is correlated with increased frequency of postoperative complications and death. In our study population, post-operative RCC demand led to significantly increased inpatient time. Further studies are needed to confirm these findings, which could implicate necessity of more attentive evaluation of PAT patients for TKA and consideration of suitability for fast track total joint arthroplasty programs. In addition, the factual necessity for antithrombotic therapy should be re-evaluated thoroughly in each case prior to intervention or TKA should be postponed until the indication for PAT has expired, if this is reasonable.

## Additional Information

**How to cite this article**: Leitner, L. *et al*. Impact of preoperative antithrombotic therapy on blood management after implantation of primary total knee arthroplasty. *Sci. Rep.* **6**, 30924; doi: 10.1038/srep30924 (2016).

## Figures and Tables

**Table 1:** Characterization of patients with longtime PAT.

Drugs	n (%)	Dosage	Length of use (months, mean ± SD)	Time since last use (days, mean ± SD)
*all PAT*	*82 (100)*	—	*51.9* ± *34.1*	*9.5* ± *5.1*
Aspirin	44 (53.7)	100 mg	49.1 ± 33.8	8.9 ± 4.0
Clopidogrel	2 (2.4%)	75mg	19.0 ± 5.0	7.5 ± 0.5
Phenprocoumon	31 (37.8)	—	58.9 ± 36.7	10.8 ± 6.5
Acenocoumarol	4 (4.9%)	—	78 ± 42.4	6.5 ± 1.0
Enoxaparin	1 (1.2%)	—	Not documented	Not documented
**Underlying disease for PAT**	**n (%)**	**needed RCC (yes, no; p-value)**		
*Cardiac*				
CVD	40 (35.7)	12, 28; p = 0.09		
MI history	14 (12.8)	5, 9; p = 0.26		
Cardiac arrhythmia	24 (22.0)	4, 20; p = 0.62		
Implanted pacer	4 (3.6)	1, 3; p = 0.82		
*Others*				
Stroke/TIA history	8 (7.3)	7, 1; p = 0.57		
Thrombosis/PE history	19 (17.4)	6, 13; p = 0.21		

Others: Acenocoumarol, clopidogrel, enoxaparin. CVD, cardiovascular disease; TIA, transient ischemic attack; PE, pulmonic embolism; RCC, red blood cell concentrate.

**Table 2:** Comparison of patients with longtime PAT (PAT patients) and without (non-PAT patients).

	PAT-Patients (n = 82)	non-PAT-Patients (n = 118)	p-Value
Patient demographics			
Age at surgery (years)	73.5 ± 5.9	70.0 ± 8.9	**0.002**
Male/female	35/47	38/79	0.17
BMI	30.6 ± 4.2	29.5 ± 5.6	0.17
ASA > 2 (yes/no)	76/6	65/53	**<0.001**
Operation			
Preoperative Hb (mg/dl)	13.8 ± 1.9	14.1 ± 1.2	0.28
Intraoperative Hb (mg/dl)	9.6 ± 1.7	10.3 ± 1.3	**0.002**
Duration of OP (min)	93.1 ± 25.8	87.6 ± 24.4	0.13
Enoxaparin dose (mg/dl)	54.2 ± 14.4	43.4 ± 7.5	**<0.001**
Post-operative			
1st day wound drain (ml)	367 ± 230	327 ± 213	0.25
RCC demand (yes/no)	26/56	15/103	**<0.001**
Average demand of RCCs	0.7 ± 1.0	0.3 ± 0.7	**0.001**
Inpatient time (days)	9.1 ± 3.7	8.7 ± 2.6	0.43

PAT, preoperative anticoaguation therapy; Hb, hemoglobin-level; OP, operation; RCC, red blood cell concentrate.

**Table 3:** Comparison of patients with postoperative demand for RCC (RCC patients) and patients without (non- RCC patients).

	RCC-Patients (n = 41)	non-RCC-Patients (n = 159)	p-Value
Age at surgery (years)	74.8 ± 7.5	70.5 ± 7.8	**0.002**
Male/female	9/32	65/94	**0.025**
BMI	29.7 ± 5.1	30.0 ± 5.2	0.70
ASA > 2 (yes/no)	34/7	107/52	0.05
PAT (yes/no)	25/16	57/102	**<0.004**
Preoperative Hb (mg/dl)	12.6 ± 1.2	14.3 ± 1.5	**<0.001**
Intraoperative Hb (mg/dl)	8.5 ± 0.7	10.4 ± 1.4	**<0.001**
Duration of OP (min)	94.6 ± 27.6	90.0 ± 24.7	0.30
Enoxaparin dose (mg/d)	50.9 ± 14.2	47.8 ± 11.9	0.20
1^st^ day wound drain (ml)	368 ± 229	339 ± 219	0.51
Inpatient time (days)	9.8 ± 4.3	8.6 ± 2.6	**0.042**

PAT, preoperative antithrombotic therapy; Hb, hemoglobin-level; OP, operation; RCC, red blood cell concentrate.

**Table 4:** PAT, preoperative antithrombotic therapy; OP, operation; BMI, body mass index.

Multivariate regression model on RCC-demand following TKA in all patients			
	Odds Ratio	95% confidence interval	p-Value
Female sex	0.96	0.83–1.09	0.088
Preoperative Hb (mg/ml)	0.90	0.86–0.94	**<0.000**
PAT	1.17	1.05–1.29	**0.005**
Age at surgery (years)	1.01	1.00–1.02	**0.014**
BMI	0.99	0.98–1.01	0.400
ASA > 2 (yes/no)	1.02	0.90–1.16	0.727
Duration of OP (min)	1.01	1.00–1.01	0.100
